# Pneumocystis Pneumonia in Locally Advanced Breast Cancer Despite Prophylactic Use of Trimethoprim-Sulfamethoxazole During Prednisolone Treatment for a Pembrolizumab-Induced Immune-Related Adverse Event: A Case Report

**DOI:** 10.7759/cureus.56868

**Published:** 2024-03-25

**Authors:** Yudai Kaneda, Kenji Gonda, Takanori Asakura, Masahiro Wada, Toyoaki Sawano, Tomohiro Kurokawa, Kazunoshin Tachibana, Akihiko Ozaki

**Affiliations:** 1 School of Medicine, Hokkaido University, Sapporo, JPN; 2 Department of Breast Surgery, Jyoban Hospital, Iwaki, JPN; 3 Division of Pulmonology, Kitasato University Hospital, Tokyo, JPN; 4 Department of Surgery, Jyoban Hospital, Iwaki, JPN

**Keywords:** stevens-johnson syndrome (sjs), prophylaxis, trimethoprim-sulfamethoxazole (tmp-smx), breast cancer, pneumocystis pneumonia (pcp)

## Abstract

Pneumocystis pneumonia (PCP) primarily affects immunosuppressed patients, with trimethoprim-sulfamethoxazole (TMP-SMX) commonly used for prophylaxis. However, there is insufficient information on PCP occurrence despite TMP-SMX prophylaxis. We encountered a 57-year-old woman with locally advanced breast cancer developing PCP despite prophylactic intake of TMP-SMX, during treatment with prednisolone for Stevens-Johnson syndrome (SJS) induced by pembrolizumab. This case underscores the need to pay attention to the possibility of PCP development even during TMP-SMX prophylaxis. Dosage and duration adjustments according to the patient's condition and weight may be required.

## Introduction

Pneumocystis pneumonia (PCP) is a severe condition predominantly found in immunocompromised individuals, with a less than 5% prevalence in most risk groups [1]. Although breast cancer is not typically linked to immunosuppression, reports have indicated an increased risk of PCP incidence in patients undergoing high-dose chemotherapy or receiving corticosteroids [2-6]. The prognosis for breast cancer patients who develop *Pneumocystis jirovecii* pneumonia can be severe, with a mortality rate of 40% reported in a study of patients with solid tumors, including breast cancer, making prevention crucial [3]. 

In this context, prophylactic treatment with trimethoprim-sulfamethoxazole (TMP-SMX) is generally recommended as a first-line regimen for prophylaxis of PCP [7]. However, an important fact to consider is that breakthrough episodes of PCP can still occur in patients who have adhered to their prophylaxis regimens [8]. Especially, immunocompromised patients are at higher risk of breakthrough PCP despite prophylaxis, with a systematic review and meta-analysis reporting a pooled incidence of breakthrough PCP with intravenous pentamidine prophylaxis (IVP) of 0.7% [9]. Although low-dose TMP-SMX regimens are reported to be effective in preventing and reducing the incidence of PCP infections and related mortality [10], the use of TMP-SMX prophylaxis in high-risk patients, such as those with rheumatic diseases exposed to prolonged high-dose glucocorticoids, is also reported to reduce those risks, and there are no solid criteria for this [11].

Here, we report on a case of recurrent PCP despite having taken prophylaxis during treatment for breast cancer.

## Case presentation

A 57-year-old female initially presented with swelling of the right breast and a palpable mass. A subsequent core needle biopsy confirmed the diagnosis of infiltrating ductal carcinoma in the right breast. The tumor, measuring 20 x 10 cm, was fixed to the chest wall and presented with significant erythema, classified as T4N1M0 stage IIIB. The cancer was characterized by being estrogen receptor (ER) negative, progesterone receptor (PgR) negative, and human epidermal growth factor receptor 2 (HER2) negative, and it had a Ki-67 proliferation index of less than 10%. In terms of her medical history, the patient had been treated for bipolar disorder, with ongoing medication including clonazepam and clotiazepam. There was no history of rheumatoid arthritis noted.

**Figure 1:**
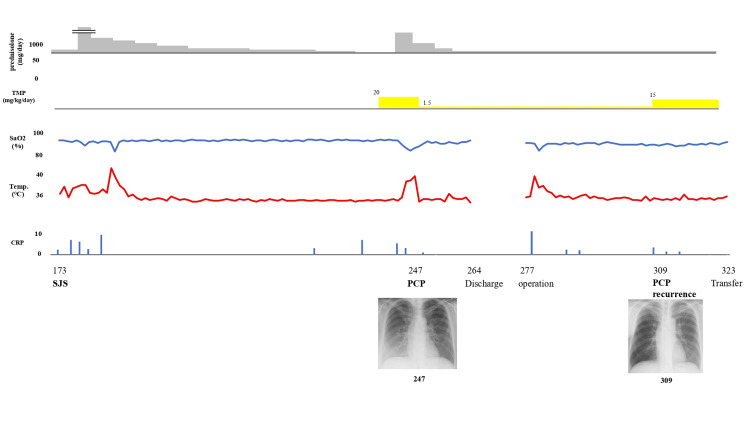
Overall picture of the patient's progress. PCP: Pneumocystis pneumonia; TMP: trimethoprim.

For locally advanced breast cancer, which is inoperable at the initial presentation, treatment with dose-dense adriamycin and cyclophosphamide (AC) therapy (doxorubicin 60 mg/m² d.i.v. day 14, cyclophosphamide 600 mg/m² d.i.v. day 14) was initiated on day seven for three cycles. During treatment, the patient needed to be hospitalized for management due to worsening psychiatric symptoms, but since this happened after the administration of the chemotherapy at the second cycle (day 21) and before the inception of the third cycle (day 36), there was no interruption of AC therapy. After the discharge and mitigation of her psychiatric symptoms, on day 140, treatment with PEM+CDCBA+PTX therapy (pembrolizumab 200 mg/body 30 min d.i.v. day one, paclitaxel 80 mg/m² d.i.v. days one, eight, 15, carboplatin AUC 5 d.i.v. day one) was administered for one cycle. During this period, the following medications were prescribed and administered from our department until day 165: famotidine 10 mg once daily, celecoxib 100 mg four times daily, olanzapine 2.5 mg twice daily, three tablets of *Clostridium butyricum*, and loperamide 1 mg two capsules twice daily. On day 167, the patient presented with fever, oral mucosal rash, facial erythema, and desquamation. She was transported to the dermatology department of a nearby general hospital, where a skin biopsy was performed on day 173 due to suspicion of immune-related adverse events from pembrolizumab. The biopsy showed vital changes at the interface of the epidermis, with scattered necrosis of the keratinocytes, leading to a diagnosis of Stevens-Johnson syndrome (SJS). Treatment with steroids (methylprednisolone (PSL) 15 mg/day) and antibiotics (cefazolin 2 g/day) were administered without improvement, leading to her transfer to a university hospital. On day 179, steroid pulse therapy (PSL 1000 mg/day) was initiated, followed by a gradual tapering, as shown in Figure 1.

**Figure 2:**
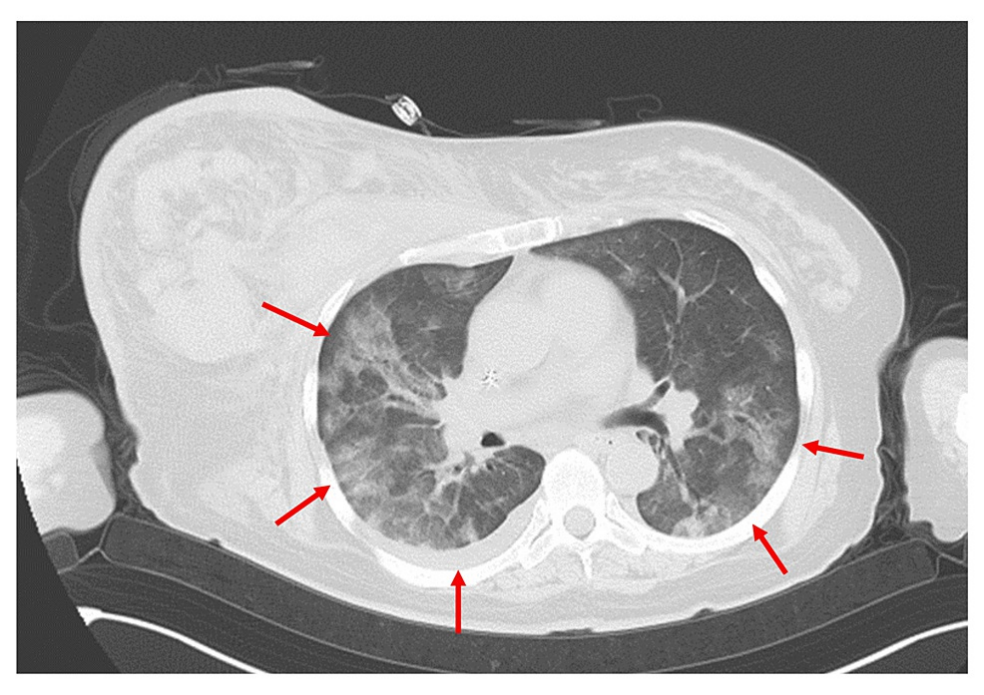
CT image of the patient on day 247. Bilateral diffuse ground-glass opacities were observed in the pulmonary fields (arrows).

On the morning of day 247, the patient experienced difficulty breathing, and a CT scan revealed bilateral diffuse ground-glass opacities in the lungs, sparing the subpleural areas (Figure 2). Additionally, elevated β-D-glucan levels (169 pg/mL) were noted in a single blood sampling. Although bronchoalveolar lavage (BAL), necessary for a definitive diagnosis, could not be performed due to the patient's overall condition, we concluded that these findings largely met the criteria for a probable diagnosis, thereby making it reasonable to diagnose the patient with PCP [12]. Treatment with TMP-SMX (trimethoprim 80 mg, sulfamethoxazole 400 mg) at 20 mg/kg/day (based on trimethoprim) was initiated orally, along with concurrent administration of PSL 80 mg/day for five days, 40 mg/day for another five days, 20 mg/day for a further five days, and after that continued at 10 mg/day.

**Figure 3:**
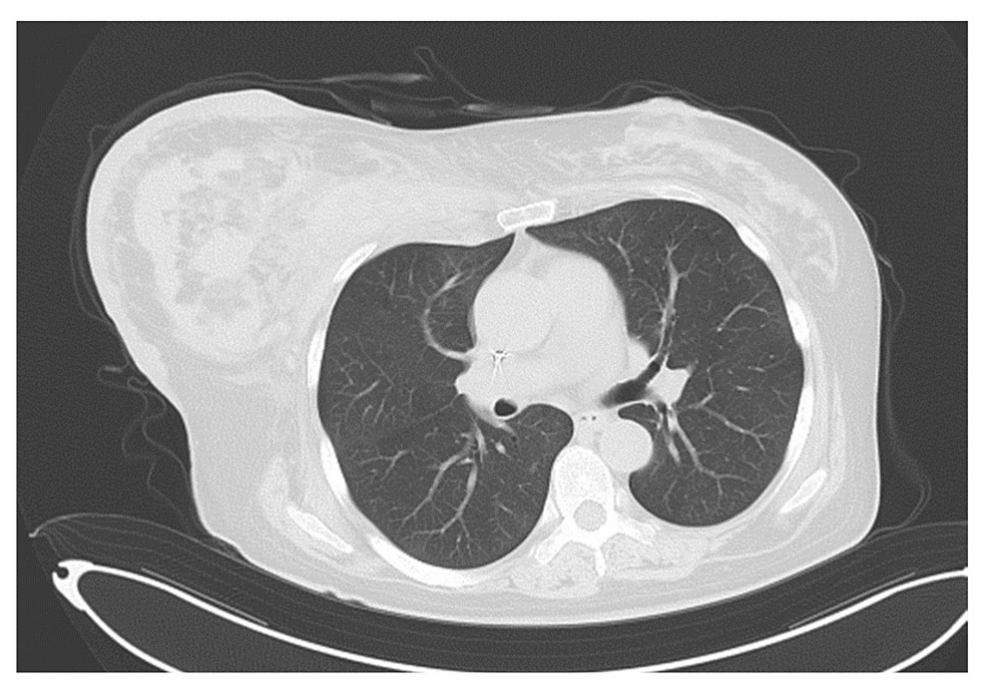
CT image of the patient on day 256. The ground-glass opacities previously identified on computed tomography at day 247 are no longer apparent.

A CT scan on day 256 showed almost complete resolution of the bilateral ground-glass opacities (Figure 3), and blood tests indicated that CRP (0.21 mg/dL) was within the normal range. However, on the same day, there were indications of liver dysfunction, with aspartate aminotransferase (AST) (66 U/L), alanine transaminase (ALT) (76 U/L), and gamma-glutamyl transpeptidase (γ-GTP) (153 U/L) elevated, as well as alkaline phosphatase (ALP) (229 U/L) and lactate dehydrogenase (LDH) (908 U/L), leading to a reduction of the TMP-SMX dose to 1.5 mg/kg/day for prophylactic continuation. The patient's condition stabilized and progressed favorably after that.

On day 277, the patient underwent a mastectomy of the breast, pectoralis primary muscle resection, axillary lymph node dissection, latissimus dorsi muscle skin flap, and split-thickness skin grafting. Although the split-thickness skin graft experienced some detachment, requiring intervention postoperatively, the patient's condition generally stabilized and progressed well afterward.

**Figure 4:**
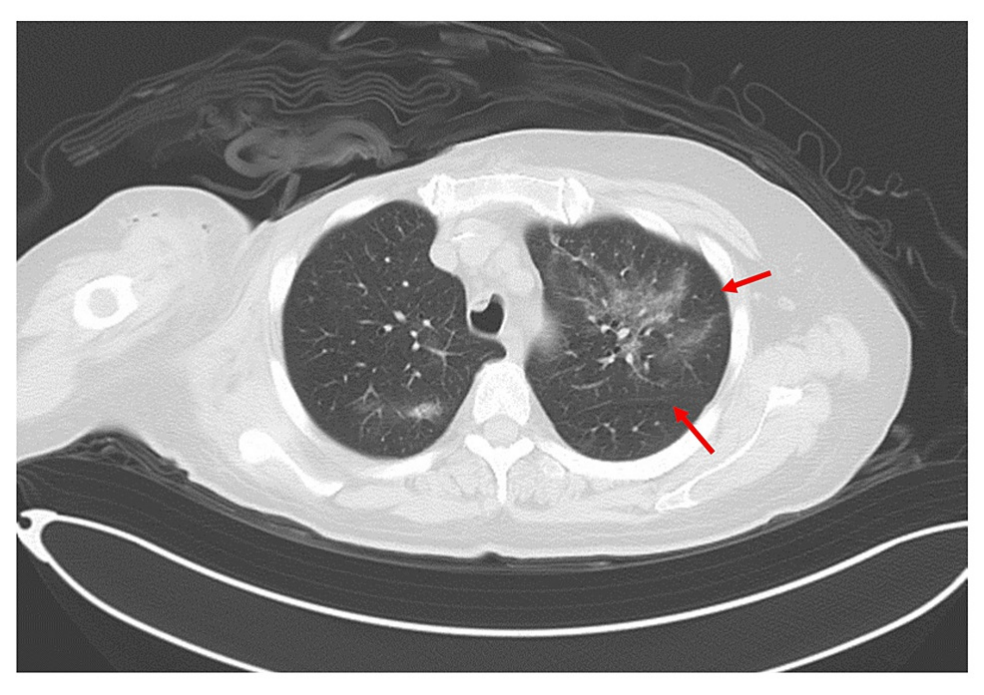
CT image of the patient on day 289. Ground-glass opacities were noted within the S1-2 segments of the left upper pulmonary lobe (arrows).

On day 289, a chest CT scan was performed to evaluate the status of the post-operative wound. Incidentally, ground-glass opacities were identified in the S1-2 segments of the left upper lobe (Figure 4). At that time, the patient was asymptomatic, and having received prophylactic doses of TMP-SMX, the findings were judged to be not indicative of PCP. Subsequently, the patient developed symptoms, including cough and dyspnea, which progressively worsened.

**Figure 5:**
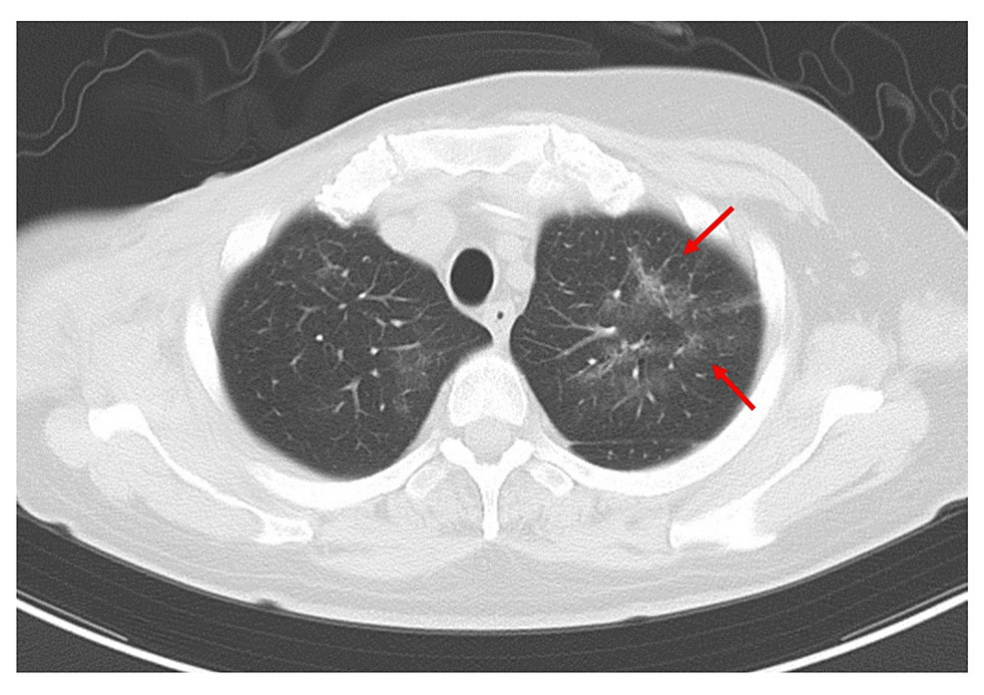
CT image of the patient on day 309. Compared with the imaging findings on day 289, the ground-glass opacities within the S1-2 segments of the left upper lobe were less conspicuous (arrows).

A follow-up CT scan on day 309 revealed persistent ground-glass opacities in the same regions of the left upper lobe, though to a lesser extent compared with the previous scan on day 289 (Figure 5). Despite an improved imaging study, given the clinical presentation, a relapse of PCP was suspected, and the dosage of TMP-SMX was increased to a therapeutic level of 15 mg/kg/day (based on the trimethoprim component). Following this intervention, the patient swiftly demonstrated clinical improvement, supporting the diagnosis of PCP.

On day 323, the patient experienced a relapse of depression, necessitating emergency psychiatric hospitalization. After that, there was no recurrence of PCP, but on day 410, the patient died due to the progression of the breast cancer.

## Discussion

PCP, despite prophylaxis, remains an area of clinical concern [13,14], as elucidated by the presented case of a 57-year-old woman undergoing treatment for breast cancer. This prompts a critical examination of the adequacy of prophylactic dosage of TMP-SMX for PCP, the reason for this occurrence, and the early signs that healthcare professionals should be attentive. Especially our case underscores the importance of individualizing the prophylaxis regimen with TMP-SMX, given that the patient experienced a recurrence. While TMP-SMX is commonly endorsed for PCP prophylaxis in breast cancer patients undergoing chemotherapy [15], and the general recommendation for PCP prophylaxis in adults, including cancer patients, is a single-strength or double-strength tablet once daily [16]. Furthermore, while it is generally reported that relapses of PCP often occur in cases of poor adherence to prophylactic medication [8], considering that adherence was maintained, in this case, the optimal usage, particularly in individuals with distinct clinical profiles, warrants a more thorough exploration [4,5]. To tailor PCP prophylaxis management individually, further exploration of information, including the search for additional relevant factors, is necessary.

Further, several reasons could explain why this patient developed PCP despite adherence to prophylaxis. Firstly, the patient's underlying malignancy and the intensive chemotherapy regimens could have further suppressed her immune system [17,18], making her more susceptible. While breast cancer itself is not easily linked to immunosuppressive conditions considering the relatively sustained efficacy of vaccination against the COVID-19 breakthrough infection [19], a regimen containing adriamycin and cyclophosphamide has been linked to PCP occurrence [3]. Furthermore, while the initial PCP episode was treated, there is a possibility of latent microorganisms that could have been activated under certain conditions. Additionally, other factors like co-existing illnesses or medication interactions, especially considering her bipolar disorder and ongoing medication, might have influenced her susceptibility.

The subtle and varied presentation of PCP, especially in patients on prophylaxis or those with other medical conditions, makes its early detection challenging [20]. In this case, the recurrence was identified with the presentation of a nocturnal cough. It underscores the need for clinicians to maintain a high degree of suspicion even in the presence of non-specific symptoms. Regular monitoring of biomarkers such as β-D-glucan, LDH, KL-6, and CRP, though within the normal range in this recurrence, might aid in early detection [14,21]. An understanding of individual patient risk, based on their medical history and treatments, will guide the frequency and intensity of this monitoring. Specifically, given the grave consequences of a missed or late diagnosis of PCP, the presence of any respiratory symptoms in such high-risk individuals should be meticulously investigated [20,22].

## Conclusions

In conclusion, this case highlights the persistent challenge of PCP recurrence despite prophylaxis in breast cancer patients. While TMP-SMX remains a staple in prophylaxis, the adequacy of its dosage, individualized patient risk factors, and vigilant monitoring of subtle signs are paramount for optimal patient outcomes. Further studies and consensus guidelines may be instrumental in delineating prophylaxis recommendations more clearly, especially in patients with unique clinical scenarios, as presented here.
